# Pandemic-Induced Disruption and the Adaptive Resilience of the Medical Supply Chain: A Case Study of Saudi Arabia

**DOI:** 10.7759/cureus.104419

**Published:** 2026-02-27

**Authors:** Omar Darkhabani, Abdalla Ahmed

**Affiliations:** 1 Import Management, Al Nahdi Medical Company, Jeddah, SAU; 2 Medical Microbiology, Noor Biotech, Khartoum, SDN

**Keywords:** centralized procurement, covid-19 pandemic response, logistics digitization, medical supply chain resilience, public-private partnership, saudi arabia

## Abstract

The COVID-19 pandemic exposed profound vulnerabilities in global medical supply chains, largely driven by a reliance on Just-In-Time (JIT) efficiency and geographically concentrated manufacturing. While many Western economies faced sustained shortages and distribution chaos, the Kingdom of Saudi Arabia (KSA) achieved a rapid transition from initial scarcity to a surplus of essential supplies. This report analyzes the Saudi Arabian experience as a successful hybrid resilience model that synthesized centralized government authority with decentralized private sector agility. Centrally, the National Unified Procurement Company (NUPCO) utilized unified purchasing power to secure international deals, while the Saudi Food and Drug Authority (SFDA) provided regulatory agility by fast-tracking imports and registrations. Complementing this, major private distributors like Al Nahdi Medical Company reported digital logistics investments enabling distribution efficiency. The study concludes that Saudi Arabia's success stemmed from the ability to rapidly pivot from cost-efficiency to security and speed operations. For future preparedness, the report advocates for a paradigm shift that prioritizes localization of manufacturing, supply chain diversification, and the integration of advanced technologies like AI and blockchain to ensure long-term national health security.

## Introduction and background

The systemic shock of COVID-19 on medical supply chains

Contextualizing the Crisis and the Research Gap

The onset of the COVID-19 pandemic imposed immense strain on global medical supply chains, immediately exposing critical, long-standing vulnerabilities due to an unprecedented surge in demand coupled with widespread disruptions to global manufacturing and logistics networks [1]. Initial global shortages were acute, affecting not only personal protective equipment (PPE) such as surgical masks, respirators, and gloves, but also critical supplies like testing reagents and essential pharmaceuticals [1]. This crisis underscored how systemic risks within the supply network gravely threaten the fundamental objective of healthcare delivery: providing effective, timely, and safe care [2].

A crucial observation during this period was the variation in national responses and recovery trajectories. While many developed economies experienced prolonged competitive bidding, chronic shortages, and distribution chaos, the experience in the Kingdom of Saudi Arabia (KSA) presented a paradox. Eyewitness accounts from KSA described an initial, short-term scarcity of items like face masks, latex gloves, and hand sanitizers, followed by a remarkably rapid government and private sector recovery that resulted in a substantial "flood" of these items into the local market.

The disparity between KSA’s quick stabilization and the sustained crises seen elsewhere suggests a critical area for investigation. This report asserts that the resilience demonstrated by the Saudi Arabian medical supply chain stemmed from a synthesized approach, combining centralized governmental policy, specifically, the regulatory agility of the Saudi Food and Drug Authority (SFDA) and the unified procurement power of the National Unified Procurement Company (NUPCO), with advanced, decentralized private sector operational digitization, exemplified by companies like Al Nahdi Medical Company. This coordinated public-private model enabled a superior speed and scale of response compared to the fragmented, purely market-driven systems that failed in many Western economies [3].

## Review

Theoretical frameworks of supply chain vulnerability in healthcare

The Conflict Between Efficiency-Driven Models and Resilience

The global shortage crisis of 2020 was fundamentally rooted in the conflict between supply chain efficiency, often mandated by Just-in-Time (JIT) principles, and resilience, which necessitates Just-in-Case (JIC) inventory buffers. Dysfunctional costing models prevalent in hospital operating systems worldwide incentivized the minimization of inventory and overhead costs, thereby placing the short-term profit motive ahead of public health preparedness [3]. This emphasis on lean operations rendered the entire medical products industry highly susceptible to major disruption. The tightly coupled production arrangements challenged the capacity to surge output, especially when non-substitutable resources, such as melt-blown, non-woven polypropylene material essential for N95 respirators and surgical masks, became scarce [2].

The crisis demonstrated that the global system had actively prioritized cost minimization over necessary redundancy, meaning that external regulation is crucial to force strategic supply chain preparation. When a resource is critical and production is geographically concentrated, the lean industrial structure becomes a systemic risk, confirming that the strategic decision to manage resource uncertainty was made in favor of cost-cutting rather than resiliency.

Resource Dependence Theory (RDT) and Geopolitical Risk

RDT offers a suitable framework for examining firms' external dependencies on essential resources, especially during periods of high uncertainty, like a pandemic [4]. The extreme concentration of manufacturing for key medical inputs, such as medical gloves and generic drugs, in single geographic areas like China and India introduced severe logistical and geopolitical risks [2]. This dependency created structural vulnerabilities that could be easily disrupted by health crises, political actions, or natural disasters.

Furthermore, a foundational lesson of the crisis is that health must be treated as a public good. When public health is at stake, market prices are inherently inappropriate mechanisms for rationing essential inputs like PPE. The inability of the market mechanism to optimally allocate these resources confirms that market failure occurred, necessitating robust governmental control over distribution and inventory to achieve optimal public outcomes rather than monetary gain [3].

Historical analysis of pandemic-driven supply constraints

Recurrent Failures in Non-pharmaceutical Intervention (NPI) Supply

The problem of mass scarcity during respiratory pandemics is not new; it is a recurrent challenge spanning over a century. The 1918 H1N1 influenza pandemic, for instance, relied heavily on NPIs due to the absence of modern vaccines and antivirals [5]. These NPIs included widespread social-distancing measures, isolation, quarantine, public gathering bans, and personal protective actions like mandatory mask ordinances [6]. This history establishes that high-mortality, rapidly spreading pandemics invariably trigger a rapid and immediate increase in demand for protective equipment, be it crude cloth masks in 1918 or advanced N95 respirators in 2020, well in excess of routine supply options. The persistence of this specific shortage pattern suggests that short-term economic optimization has perpetually overshadowed necessary long-term public health planning. Researchers argue that the healthcare supply chain has historically favored individual-level cost optimization over system-level resilience, a pattern observed during the 2009 H1N1 pandemic and repeated in 2020 [7]. Specifically, hospital budgeting models often treat PPE as a "sunk cost" or an expenditure to be minimized rather than a critical resilience asset, leading to a systemic lack of adequate inventory [3].

Geopolitical and Natural Disaster Dependency Crises

Historical disruptions underscore that single-source dependency is a fatal vulnerability. A stark example is the Quinine Crisis of 1942, where the Japanese Empire's occupation of the East Indies eliminated 90% of the world's supply of quinine, the most effective antimalarial drug of the era [8]. This extreme geopolitical disruption demonstrated how non-health crises can completely destroy access to essential medical supplies if sourcing is concentrated.

More recently, the impact of localized natural events was highlighted in 2017 when Hurricane Maria devastated Puerto Rico, leading to a severe shortage of intravenous (IV) saline across the United States [8]. This illustrates that despite technological advances, modern, geographically concentrated medical supply chains remain acutely vulnerable to disruption, reinforcing the core tenets of RDT regarding external resource dependencies. The repeated confirmation of scarcity across a century of major health and geopolitical events confirms the mandate for diversified and decentralized sourcing strategies [9].

Global failures in the COVID-19 era (2020-2021)

Production Bottlenecks and Trade Protectionism

The initial phase of the COVID-19 pandemic was defined by severe systemic failures in the global supply chain governance. China, the dominant global manufacturer of PPE, rapidly shifted its policy, increasing imports while decreasing exports of essential personal protective equipment [10]. This concentrated supply action removed considerable quantities of PPE from global markets, inducing panic among policymakers worldwide [10]. This market leverage resulted in a rapid price surge; China's export prices for PPE skyrocketed and remained highly elevated throughout 2020, reflecting the intensity of the global shortage and confirming the failure of market mechanisms to allocate essential resources appropriately [10].

Adding complexity, pre-existing trade war tariffs implemented by countries like the US contributed to higher prices and lower availability of PPE imports from China immediately before and during the crisis, demonstrating a failure of integrated national policy planning [3]. The crisis environment confirmed that while internationalization itself is not the root cause of shortages, it served as a primary mechanism for the rapid transmission of supply shocks, exacerbating interdependence between manufacturing and consuming economies [1]. The fragility of the system was amplified by these policy actions, creating a destructive, self-reinforcing cycle of scarcity and high cost.

Failure in Distribution Governance

A defining characteristic of the crisis in many countries was the failure of governmental institutions to maintain and efficiently distribute adequate domestic inventories, which amplified the scarcity problem [3]. This governance failure led to chaotic, competitive procurement practices among various entities-hospitals, local governments, businesses, and private individuals-making the acquisition of PPE costly and inefficient [3]. This lack of centralized, effective distribution governance contributed significantly to the global market failure observed (Table 1).

**Table 1:** Structural vulnerabilities and amplifying factors in global COVID-19 PPE supply chains (2020) References: [2,3] JIT: Just-in-Time; PPE: personal protective equipment

Vulnerability Mechanism	Root Cause	Pandemic-Era Consequence
Lean Inventory/JIT Mandates	Profit-driven hospital costing models disincentivize stockpiling	Depleted domestic inventories; competition among buyers
Geographic Concentration	Reliance on single/few regions (e.g., China) for bulk manufacturing	Export restrictions caused global market panic and price spikes
Market Pricing Mechanism	Treatment of health inputs as ordinary commodities	Prices soared; "panicked marketplace behavior" and hoarding depleted stocks
Governmental Fragmentation	Failure to maintain and distribute national stockpiles	Ineffective distribution, procurement inefficiency, and competitive bidding

Case study: the Saudi Arabian response and rapid stabilization

Centralized Procurement as a Rapid Mitigation Tool

The rapid stabilization observed in KSA can be traced directly to the synergistic actions of its centralized procurement and regulatory bodies. The NUPCO, which centralizes procurement for the medical sector [11], played a pivotal role. Although NUPCO’s traditional lowest-price tendering policies have historically been implicated in contributing to some drug shortages [11], its structure allowed it to function as a singular, powerful national buyer during the acute crisis phase.

This centralized purchasing power enabled NUPCO to "seamlessly conclude deals" for vital equipment, such as COVID-19 testing kits, early in the pandemic [11]. By acting as a unified entity, KSA effectively circumvented the destructive internal and external competitive chaos witnessed in fragmented systems. Furthermore, while the NUPCO tender process typically secures the lowest price, the flexibility existed for public institutions to resort to direct purchasing from the market at higher, regulated SFDA prices when initial supplies were exhausted. This flexibility prioritized speed of acquisition over cost containment during the immediate emergency [11].

SFDA Regulatory Agility and Import Fast-Tracking

The government's ability to transition rapidly from scarcity to surplus was heavily dependent on regulatory streamlining. The SFDA responded by demonstrating exceptional administrative agility. The SFDA swiftly expedited the import, registration, approval, and distribution processes for essential PPE, test kits, and pharmaceuticals [12]. This proactive regulatory stance was a critical mechanism that enabled the observed "flood" of supplies. It ensured that successfully secured international shipments were not stalled by local clearance procedures, thereby protecting the public and enabling swift transmission mitigation [12].

The success of the KSA model was thus defined by centralized coordination: NUPCO secured the global supply, and the SFDA secured the legal and logistical rapid transit domestically. This public-sector synergy created the necessary environment for subsequent efficient private-sector execution. The experience confirms that the most effective national policy in a pandemic is one that can rapidly shift from cost-efficient operations to security-and-speed operations using existing centralized structures, demonstrating that policy flexibility is paramount to national recovery.

The role of major private distributors: operational resilience of Al Nahdi Medical Company

Strategic Digital Transformation and Logistics Optimization

The public sector’s success in securing the supply needed to be matched by the private sector’s ability to distribute it rapidly. Major distributors, such as Al Nahdi Medical Company, recognized the limitations of traditional, manual logistics in handling the non-linear demand surge. To modernize fulfillment and streamline delivery, Al Nahdi Medical Company strategically invested in advanced systems, implementing Oracle Transportation Management and Oracle Warehouse Management solutions [13].

This dedication to technological readiness directly translated into high operational resilience. The digitization of the logistics chain enabled Al Nahdi to achieve efficiency gains of "almost five times" that of its old distribution center, resulting in maintained availability of over 95% across its consumer touchpoints [13].

The Private Sector’s Role in Supply Velocity

Beyond a single company, the broader Saudi healthcare supply chain management (SCM) witnessed enhanced efficiency post COVID-19 through system-wide adoption of new technologies like AI, blockchain, and the Internet of Things (IoT) [14]. These technologies provided increased traceability, greater visibility, and updated forecasting capabilities, which are essential components for smarter inventory management and enhanced agility [14].

The digitized supply chain thus served as a force multiplier for the government's procurement success. Without the reported efficiency gains and real-time visibility [13], the procured "flood" of supplies would have stagnated in warehouses, leading to continued localized shortages despite national inventory availability. The efficiency gains observed (5x throughput and >95% availability) were transformative, providing the non-linear processing capacity required to match the non-linear demand shock. This confirms that public health preparedness mandates technological readiness in critical private logistics infrastructure [15]. Large distributors also contributed to resilience by actively broadening sourcing relationships beyond geographically concentrated origins, promoting supply chain diversity (Table 2) [16].

**Table 2:** Operational resilience metrics and outcomes in Saudi Arabian private logistics (example: Al Nahdi Medical Company) References: [12,13]

Resilience Domain	Pre-crisis Condition (Implied)	Crisis Response Metric (2020-2021)	Enabling Technology/Strategy
Inventory availability	Optimized for cost efficiency (lower margins)	Maintained >95% availability across guest touchpoints	Oracle Warehouse Management
Distribution efficiency	Standard logistics velocity	Achieved 5x operational efficiency gains	Oracle Transportation Management
Supply chain visibility	Traditional/Fragmented tracking	Real-time visibility and compliance achieved	Cloud supply chain management, AI, the Internet of Things, and blockchain adoption
Regulatory compliance	Manual processes	Expedited import and distribution compliance	Saudi Food and Drug Authority fast-tracking, coupled with real-time data

Discussion and policy implications for future preparedness

The KSA Model: A Comparative Critique of Market Fragmentation

The rapid market correction in KSA provides compelling evidence that centralized strategic governance is an essential requirement during an acute, immediate crisis. This structure proved highly effective in preventing the destructive internal competition and severe price gouging that afflicted fragmented market-driven systems [3]. The strategic coordination between NUPCO and the SFDA in expediting imports [12] established a blueprint for regulatory responsiveness, illustrating that bureaucratic inertia itself constitutes a major latent safety problem in emergencies [2].

Strategies for Sustainable Resilience: Localization and Diversification

The consensus following COVID-19 mandates a fundamental shift away from prioritizing JIT cost-efficiency toward prioritizing strategic persistence and flexibility [17]. For nations like KSA, this means aggressively pursuing the industrial policy goals laid out in Vision 2030, specifically the localization of pharmaceutical manufacturing. Future policy must pursue strategic industrial policy to reduce reliance on imported PPE [3], requiring governmental incentives and regulations to strengthen domestic production capabilities. Localization must be comprehensive, addressing raw material security and skilled labor availability, and establishing operations closer to target markets to reduce lead times and transport costs [18]. Furthermore, diversifying supply chain relationships is crucial for mitigating RDT risks, moving past single-source dependencies to ensure resilience.

Governance and Transparency in the Post-pandemic Era

Achieving robust, long-term resilience requires a new governance structure that integrates public and private sectors, forming a "commons" to oversee and direct activity [17]. This system must prioritize five key attributes: flexibility, traceability and transparency, persistence, global independence, and equity in distribution [17]. Such a structure should oversee major purchase orders, distribution of critical supplies, and strategic expansion of national production capacity.

Policy recommendations arising from this analysis include mandating a conducive and flexible regulatory environment [19] and requiring the healthcare industry to regularly stress-test its supply systems to ensure their capacity to withstand future disruptions. For preparedness, the supply chain must be managed not merely as an economic function, but as a foundational national security infrastructure.

## Conclusions

The COVID-19 pandemic served as a pivotal stress test, exposing long-standing vulnerabilities in global medical supply chains rooted in geographic dependency and economic models that rigidly prioritize short-term cost savings over long-term strategic resilience. This structural fragility, exacerbated by geopolitical trade restrictions and fragmented government responses, resulted in widespread global shortages. In contrast, the Saudi Arabian experience of moving rapidly from scarcity to surplus confirms the effectiveness of a hybrid resilience model. This model successfully married centralized regulatory authority (SFDA) and strategic unified procurement (NUPCO) with highly advanced, technologically driven private sector distribution agility (exemplified by Al Nahdi Medical Company). This unique blended public-private framework offers critical lessons for global health security planning.

The mandate for future preparedness is clear: transformative changes are necessary. Policy must shift governance models toward mandating diversification, transparency, and significant technological investment in SCM infrastructure. For nations reliant on imports, aggressive industrial policy aimed at localization and strategic stockpiling is the essential mechanism for ensuring that the supply chain can transition rapidly and effectively from normal operation to crisis response, thereby safeguarding public health.
